# Dysfunction of inhibitory interneurons contributes to synaptic plasticity via GABABR-pNR2B signaling in a chronic migraine rat model

**DOI:** 10.3389/fnmol.2023.1142072

**Published:** 2023-05-26

**Authors:** Xiaoxu Zeng, Yingying Niu, Guangcheng Qin, Dunke Zhang, Lixue Chen

**Affiliations:** ^1^Department of Laboratory Medicine, West China Second University Hospital, Sichuan University, Chengdu, China; ^2^Key Laboratory of Birth Defects and Related Diseases of Women and Children, Sichuan University, Ministry of Education, Chengdu, China; ^3^Laboratory Research Center, The First Affiliated Hospital of Chongqing Medical University, Chongqing, China

**Keywords:** chronic migraine, inhibitory interneurons, GABABR2, NR2B, synaptic plasticity

## Abstract

**Background:**

According to our previous study, the loss of inhibitory interneuron function contributes to central sensitization in chronic migraine (CM). Synaptic plasticity is a vital basis for the occurrence of central sensitization. However, whether the decline in interneuron-mediated inhibition promotes central sensitization by regulating synaptic plasticity in CM remains unclear. Therefore, this study aims to explore the role of interneuron-mediated inhibition in the development of synaptic plasticity in CM.

**Methods:**

A CM model was established in rats by repeated dural infusion of inflammatory soup (IS) for 7 days, and the function of inhibitory interneurons was then evaluated. After intraventricular injection of baclofen [a gamma-aminobutyric acid type B receptor (GABABR) agonist] or H89 [a protein kinase A (PKA) inhibitor), behavioral tests were performed. The changes in synaptic plasticity were investigated by determining the levels of the synapse-associated proteins postsynaptic density protein 95 (PSD95), synaptophysin (Syp) and synaptophysin-1(Syt-1)]; evaluating the synaptic ultrastructure by transmission electron microscopy (TEM); and determining the density of synaptic spines via Golgi-Cox staining. Central sensitization was evaluated by measuring calcitonin gene-related peptide (CGRP), brain-derived neurotrophic factor (BDNF), c-Fos and substance P (SP) levels. Finally, the PKA/Fyn kinase (Fyn)/tyrosine-phosphorylated NR2B (pNR2B) pathway and downstream calcium-calmodulin-dependent kinase II (CaMKII)/c-AMP-responsive element binding protein (pCREB) signaling were assessed.

**Results:**

We observed dysfunction of inhibitory interneurons, and found that activation of GABABR ameliorated CM-induced hyperalgesia, repressed the CM-evoked elevation of synapse-associated protein levels and enhancement of synaptic transmission, alleviated the CM-triggered increases in the levels of central sensitization-related proteins, and inhibited CaMKII/pCREB signaling via the PKA/Fyn/pNR2B pathway. The inhibition of PKA suppressed the CM-induced activation of Fyn/pNR2B signaling.

**Conclusion:**

These data reveal that the dysfunction of inhibitory interneurons contributes to central sensitization by regulating synaptic plasticity through the GABABR/PKA/Fyn/pNR2B pathway in the periaqueductal gray (PAG) of CM rats. Blockade of GABABR-pNR2B signaling might have a positive influence on the effects of CM therapy by modulating synaptic plasticity in central sensitization.

## Introduction

Migraine is a severe neurovascular disease with repeated headache attacks as the main symptom. A minority of people with migraine develop chronic migraine (CM), which refers to a syndrome of frequent headaches on at least 15 days per month (Olesen, 2018; Buse et al., 2019). Because of the frequent attacks and high disability rates, CM imposes substantial burdens on patients, including enormous costs and reduced activity efficiency (Dodick, 2018). However, the pathogenesis of CM is still poorly understood. An accumulating body of evidence indicates that central sensitization is the underlying pathophysiological mechanism of CM (Kuner, 2010; Mathew, 2011). Central sensitization is considered to represent the increase in the excitability of neurons and the enhanced synaptic efficacy in the nociceptive pathway in response to stimuli (Yang et al., 2002; Kawasaki et al., 2008). Brain-derived neurotrophic factor (BDNF) activates intracellular metabotropic glutamate 5 receptor (mGluR5) via tyrosine receptor kinase B (TrkB)-excitatory amino acid transporter 3 (EAAT3) signal. mGluR5 is essential for enhanced Ca^2+^-dependent induction of c-Fos, a marker of neuronal activation, and plays a crucial role in the glutamate-related central sensitization underlying CM (Liu et al., 2018). SP is a key neuropeptide substance in the trigeminovascular system. Noxious stimulation-induced increases in SP expression enhance the transmission of nociceptive signals and induce hyperalgesia (Tajti et al., 2015). In previous studies by our research group, it was found that the expression of BDNF, c-Fos and SP was significantly increased in the trigeminal nucleus caudalis (TNC) of CM rats, strongly reflecting the development of central sensitization in CM (Long et al., 2020; Zhou et al., 2020).

Synaptic plasticity has been widely accepted as a vital mechanism for the development of central sensitization (Woolf and Salter, 2000; Ji et al., 2003). Synaptic plasticity refers to changes in the morphology and function of synapses, which result in enhancement of synaptic transmission efficiency. Receptor-mediated postsynaptic flow and changes in the number of neurotransmitters released from the presynaptic terminal play key roles in the formation of synaptic plasticity (Ji et al., 2003). Postsynaptic density protein 95 (PSD95) is the most abundant structural protein in postsynaptic densification in the central nervous system (Levy et al., 2015). Synaptophysin (Syp), a specific marker of synaptic plasticity, can indirectly reflect the integrity and functional state of synaptic structure (Zhu X. et al., 2019). Synaptophysin-1 (Syt-1) is a calcium-binding protein that controls the release of calcium-dependent transmitters. PSD95, Syp and Syt-1 expression has been used to provide valuable insights into synaptic plasticity. NR2B, a subunit of the ionotropic glutamate N-methyl-D-aspartate (NMDA) receptor, modulates synaptic plasticity and facilitates central sensitization through phosphorylation in CM (Wang et al., 2018). The NMDA receptor is an important receptor in the glutamate excitatory system, and one of the key mechanisms regulating NMDA receptor function involves tyrosine phosphorylation of NR2B subunits by Fyn. Tyrosine-phosphorylated NR2B (pNR2B) is the activated form of the NMDA receptor, which plays a decisive role in controlling the influx of Ca^2+^ through the NMDA receptor, regulating synaptic transmission, and determining the properties of the NMDA receptor (Moon et al., 1994; Lau and Huganir, 1995).

In the central nervous system, gamma-aminobutyric acid (GABA) is synthesized by glutamate decarboxylase 65/67 (GAD65/67) in inhibitory interneurons and is then released from the terminals of inhibitory interneurons, after which it plays a crucial role in the modulation of neuronal excitability and plasticity by enhancing gamma-aminobutyric acid type B receptor (GABABR)-mediated inhibition (Costa, 1998; Roth and Draguhn, 2012). Functional GABABR is an obligate heterodimer composed of the GABAB1 subunit (GABAB1) and GABAB2 subunit (GABAB2). Long-term potentiation (LTP) is a form of synaptic plasticity (Sanchez-Vives et al., 2021), and an inhibitory role for GABABR in the induction of LTP has been demonstrated in the CA1 area of hippocampus both *in vitro* and *in vivo* (Olpe and Karlsson, 1990; Olpe et al., 1993). In our previous studies, we found that the effect of inhibitory interneurons on central sensitization involves the regulation of GABABR on synaptic cell adhesion molecule 1 in the periaqueductal gray (PAG) (Zeng et al., 2020). Therefore, we boldly speculated that the development of central sensitization caused by the dysfunction of inhibitory interneurons may involve changes in the morphology and function of synapses.

To further clarify whether the central sensitization resulting from weakening of the function of inhibitory interneurons in CM is involved in the synaptic plasticity mechanism, we propose a hypothesis that a deficit in the function of inhibitory interneurons contributes to central sensitization by regulating synaptic plasticity through GABABR-pNR2B signaling in the PAG of CM rats. Based on the above hypothesis, we used repeated dural infusion of IS to establish a CM animal model, and the CM-associated abnormal behaviors, neurochemical variations in the PAG, and morphological changes in the PAG were explored. In this study, we indicate a new role of GABABR as a regulator of synaptic plasticity in CM and reasonably clarify the specific participation mechanism by which inhibitory interneurons participate in the modulation of synaptic plasticity in CM.

## Materials and methods

### Animals

**FIGURE 1:**
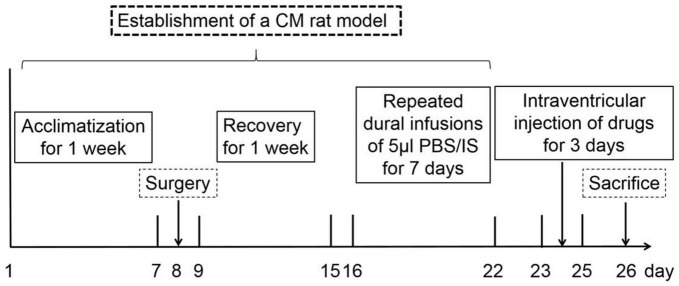
The timeline and experimental steps in this research. After 1 week of acclimatization, all the rats were randomly assigned to the experimental groups. On the 8th day, rats experienced surgery, and some rats with damaged dura were excluded. From the 9th day, rats were recovered for 1 week to ensure that the pain thresholds returned to the pre-operative level. From the 16th day, rats underwent dural infusions of 5 μl PBS/IS and behavioral tests for 7 days. From the 23rd day, rats have been treated with intraventricular baclofen or H89 injection for 3 days. On the 26th day, behavioral tests were performed, and rats were euthanized for the western blotting, HPLC, TEM, Golgi-Cox staining, and immunofluorescence staining assays.

A total of 214 healthy adult male Wistar rats (250–300 g; specific pathogen-free; certificate no. SCXK[LIAO] 2015–0001; Liaoning, China) were used to conduct experiments because of a strong correlation between migraine frequency and estrogen level (Ibrahimi et al., 2014). All animals were obtained from Liaoning Changsheng Biotechnology Co., Lt (Benxi, Liaoning, China). Rats were housed in a temperature-controlled (23 ± 1°C) room with a 12-h light/dark cycle and enough water and food. All the procedures conformed to the National Institutes of Health Guide for the Care and Use of Laboratory Animals. The experimental steps and timeline in this study are illustrated in Figure 1. All experiments performed on the animals were approved by the Ethics Committee of the Department of Medical Research (First Affiliated Hospital of Chongqing Medical University).

### CM model

The operation was performed in reference to the previous procedures (Oshinsky and Gomonchareonsiri, 2007; Melo-Carrillo and Lopez-Avila, 2013). Rats were first anesthetized with 10% chloral hydrate (4 ml/kg, intraperitoneal), then 0.01 mg/kg buprenorphine was injected subcutaneously for analgesia. A stereotaxic frame (ST-51603; Stoelting Co., Chicago, IL, USA) was used to fix the rat’s head. Then, an incision approximately 2 cm long (along the line from the midpoint of the eyes to the midpoint of the ears) was made to fully expose the skull following disinfection with iodine. A burr drill was used to carefully perform craniotomy with a diameter of 1 mm above the left dura (−1.0 mm rear from the bregma, + 1.5 mm lateral to the bregma). Next, a stainless-steel cannula with a plastic cap was fixed on the skull with dental cement. After the incision was sutured, a temperature-controlled electric blanket was provided to help the rats restore quickly. Afterward, rats were allowed to recover for a week during which the wound was disinfected daily with iodophor. Finally, rats were randomly assigned to the Sham or CM group after their pain thresholds had returned to pre-operative levels. Rats in the Sham group were infused with 5 μl phosphate-buffered saline (PBS) (0.1 M, pH 7.4) for 7 consecutive days, and Rats in the CM group were infused with 5 μl inflammatory soup (IS) for 7 consecutive days. The IS was composed of 1 mM bradykinin, 1 mM serotonin, 1 mM histamine and 0.1 mM prostaglandin E2, which were dissolved in PBS. All the above products were purchased from Sigma (St. Louis, MO, USA).

### Drug administration

To explore the roles of GABABR and protein kinase A (PKA) in CM, baclofen (MedChemExpress, Monmouth Junction, NJ, USA), a GABABR agonist, or H89 (Selleck, Houston, TX, USA), a PKA inhibitor, was dissolved in saline, and the customized dose of baclofen (0.5 μg and 5 μg) or H89 (1 μg and 10 μg) was administered intraventricularly for 3 days as described previously (Shafizadeh et al., 1997) after 7th dural infusion of PBS or IS (−1.0 mm rear from the bregma, + 1.5 mm lateral to the bregma, 4.0 mm from the skull plane; The microsyringe was used to pierce the dura mater through the cannula and reach the lateral ventricle). Depending on the experimental design, animals were randomly divided into the following 8 groups: (1) Sham group, (2) CM group, (3) CM + baclofen group, (4) Sham + baclofen group, (5) CM + H89 group, (6) Sham + H89 group, (7) CM + Saline group, (8) Sham + Saline group. In group (3), (4), (5), or (6), baclofen or H89 was injected intraventricularly in a volume of 10 μl (1 ul/min) after rats underwent dural infusion of IS or PBS. In groups (7) and (8), Rats were treated with the equivalent volume of saline intraventricularly as the control after rats underwent dural infusion of IS or PBS. The number of rats used in each group is shown in Table 1.

**TABLE 1:** Animal numbers in each group.

	Animals used							
**Experimental group**	**Behavioral tests**	**HPLC**	**WB**	**IF**	**TEM**	**Golgi-Cox**	**Mortality**	**Total**
Sham	10^a^	6	6	6	6	6	–	30
CM	10^a^	6	6	6	6	6	2	32
CM + +Saline	10^a^	–	6	6	6	6	1	25
CM + Baclofen (0.5 μg)	10	–	–	–	–	–	1	11
CM + Baclofen (5 μg)	10^a^	–	6	6	6	6	2	26
CM + H89 (1 μg)	10	–	–	–	–	–	1	11
CM + H89 (10 μg)	10	–	6^a^	–	–	–	1	11
Sham + Saline	10	–	–	–	–	–	–	10
Sham + Baclofen (0.5 μg)	10	–	–	–	–	–	1	11
Sham + Baclofen (5 μg)	10^a^	–	6	6	6	6	1	25
Sham + H89 (1 μg)	10	–	–	–	–	–	–	10
Sham + H89 (10 μg)	10	–	–	–	–	–	2	12
Total	70	12	30	30	30	30	12	214

^a^Indicates shared with other experiments, do not count.

### Behavioral tests

Both thermal and mechanical methods can be used to measure hyperalgesia. Due to the difference in bioassay sensitivity caused by the difference in noxious stimulus type, the sensitivity of the two methods is also different. Thermal devices can detect robust hyperalgesia, while mechanical devices cannot detect hyperalgesia. Therefore, the combination of thermal and mechanical methods can better monitor the hyperalgesia state. Notably, when the local thermal response was missing, it was instead measured for mechanical hyperalgesia. Prior to the first PBS/IS infusion, mechanical and thermal pain thresholds were measured as a baseline. Then, pain threshold detection was performed 24 h after each PBS/IS infusion. To evaluate the effects of the drug on the mechanical pain thresholds, we added a test after the last intraventricular drug injection. All animal behavior tests were performed under light conditions between 09:00 and 15:00 by an experimenter blinded to the experimental groups.

The mechanical threshold was used as the indicator of mechanical sensitivity, which was determined by an electronic von Frey monofilament (ElectrovonFrey, 2391, IITC Inc., Woodland Hills, CA, USA) (Wu et al., 2017). The animals were acclimated in a transparent testing box for at least 30 min. Then the tip of pressure probe was applied with increasing force to a fixed area of paw (the middle of the plantar surface of the left hind paw) (Wang et al., 2005) or periorbital region (the right and left side of the face over the rostral portion of the eye) (Romero-Reyes and Ye, 2013) of each rat. A positive response was suggested when the rat’s left hind paw or head quickly moved or retracted from the probe tip. The mechanical pain thresholds values were recorded automatically and were measured at least three times in each animal with an interval of at least 5 min. The average of the three results was considered the final mechanical pain threshold.

The thermal pain threshold was used as the indicator of thermal sensitivity, which was measured by the plantar test apparatus (Hargreaves’s test) (Techman PL-200, Chengdu, China). Thermal pain thresholds in rats were assessed by measuring the paw withdrawal latency (PWL) according to the method described previously (Hargreaves et al., 1988). The animals were acclimated in a transparent testing box for at least 30 min. Radiant heat was applied to a fixed area of each rat (the middle of the plantar surface of the right hind paw). The thermal stimulation stopped automatically when the rat lifted or licked the hind paw, and the stimulus time was recorded and considered the PWL. An automatic 30 s cutoff time was set to avoid injury to the animals. The thermal pain thresholds were measured at least three times in each animal with an interval of at least 5 min. The average of the three results was considered the final thermal pain threshold.

### Western blotting (WB)

With reference to the stereoscopic map of the rat brain, after the rats were euthanized, the rat brain was separated from the skull and placed on ice. Then, the cerebellum was removed with a blade to expose the midbrain aqueduct. Finally, the gray area around the midbrain aqueduct, the PAG, was isolated with a blade and tweezers. The PAG samples were homogenized in a radioimmunoprecipitation assay (RIPA) lysis buffer (Beyotime, Shanghai, China) with protease inhibitor PMSF (Beyotime, Shanghai, China) at 4°C for 1 h following rats were sacrificed. The protein concentration was analyzed using the Bicinchoninic Acid (BCA) protein analysis kit (Beyotime, Shanghai, China). The protein samples were electrophoresed on an SDS-PAGE gel (Beyotime, Shanghai, China), and transferred to polyvinylidene difluoride (PVDF) membrane (Millipore, USA). Next, the membranes were blocked with 5% non-fat milk for 2 h at room temperature. And the membranes were incubated overnight at 4°C with the primary antibodies diluted in TBST: anti-CGRP (1:3,000 Abcam, Cambridge, UK), anti-GAD65/67 (1:2,000 Sigma, St. Louis, MO, USA), anti-GABAB1 (1:1,000 Abcam, Cambridge, UK), anti-GABAB2 (1:1,000 Abcam, Cambridge, UK), anti-PSD95 (1:1,000 Abcam, Cambridge, UK), anti-Synaptophysin (1:5,000 Abcam, Cambridge, UK), anti-synaptotagmin1 (1: 500 Bioss, Beijing, China), anti-BDNF (1:1,000 Abcam, Cambridge, UK), anti-c-Fos (1:1,000 Abcam, Cambridge, UK), anti-PKA C-α (1:3,000 Cell Signaling Technology, Danvers, MA, USA), anti-Fyn (1:1,000 Abcam, Cambridge, UK), anti-pNR2B-Y1472 (1:500 Bioss, Beijing, China), anti-NR2B (1:1,000 Proteintech, Rosemont, IL, USA), CaMK II (1:1,000 R&D Systems, Minneapolis, MN, USA), anti-pCREB ser133 (1:3,000 Abcam, Cambridge, UK), anti-CREB (1:3,000 Cell Signaling Technology, Danvers, MA, USA), anti-GAPDH (1:5,000 Bioss, Beijing, China) and anti-β-actin (1:9,000 Proteintech, Rosemont, IL, USA). The next day, the HRP-conjugated anti-rabbit secondary antibody (1:9,000 Bioss, Beijing, China) and anti-mouse secondary antibody (1: 5,000 Zhongshan Golden Bridge Bio, China) were applied at 37°C for 1 h. Then, the membranes were revealed with the Beyo ECL Plus kit (Beyotime, Shanghai, China). The β-actin or GAPDH was used as a control to normalize protein expression.

### Immunofluorescence staining

Rats were perfused transcardially with 4% paraformaldehyde following anesthetized. To maintain integrity, the PAG tissues and their surrounding extensions were isolated fleetly after perfusion, then post-fixed in 4% paraformaldehyde at 4°C for 24 h, subsequently transferred to sucrose with increasing concentrations (20–30%) until it sank. Next, the samples were sliced transversely at 10 μm by a freezing microtome (Leica, Japan). The glass slides were used to collect and secure tissue sections. After antigen retrieval with sodium citrate (Beyotime, Shanghai, China), the sections were incubated with 10% goat serum (Boster, Wuhan, China) at 37°C for 30 min. And the sections were incubated overnight at 4°C with the primary antibodies diluted in PBS: rabbit anti-rat GAD65/67 antibody (1:100 Sigma, St Louis, MO, USA), mouse anti-rat CGRP antibody (1:50 Santa Cruz, Santa Cruz, CA, USA), rabbit anti-rat PSD95 antibody (1:500 Abcam, Cambridge, UK), rabbit anti-rat Synaptophysin (1:50 Proteintech, Rosemont, IL, USA), rabbit anti-rat synaptotagmin1 (1:100 Bioss, Beijing, China), rabbit anti-rat c-Fos antibody (1:1,000 Synaptic Systems, Germany), mouse anti-rat SP (1:50 Abcam, Cambridge, UK), mouse anti-rat GABAB2 antibody (1:50 Santa Cruz, Santa Cruz, CA, USA), and rabbit anti-rat NR2B antibody (1:50 Proteintech, Rosemont, IL, USA). The next day, Alexa Fluor 488-conjugated goat anti-mouse immunoglobulin G (IgG) (1:500 Beyotime, Shanghai, China) and Cy3-conjugated goat anti-rabbit IgG (1:500 Beyotime, Shanghai, China) were applied at 37°C for 1.5 h. Afterward, the sections were incubated with 4’,6-diamidino-2-phenylindole (DAPI) (Beyotime, China) at 37°C for 10 min. The images were captured under a confocal laser scanning fluorescence microscope (ZEISS, Germany) and the fluorescence intensity was analyzed using Image-J. The experimenters who performed the image analysis were blinded to the experimental groups.

### High-performance liquid chromatography (HPLC)

The total GABA concentration in the PAG was determined using HPLC. PAG tissues were immediately harvested after rats were sacrificed 24 h after the 7th dural infusion of PBS or IS. After weighed accurately, the PAG samples were homogenized with the internal standard solution at a weight: volume ratio of 1:1 (mg: μl), and the internal standard solution was a BABA (3-aminobutyric acid) solution with a concentration of 600 μg/mL prepared with the ultrapure water. Next, the protein was precipitated by the acetonitrile [the weight: volume ratio 1:5 (mg:μl)] and then centrifuged at 4°C (12,000 *g*, 10 min). The final samples were obtained by diluting the supernatants 10-fold. Referring to the method described previously (Liu et al., 2020), the final samples were tested by HPLC. The analytical instrument was an Agilent 1,260 series high-performance liquid chromatograph equipped with a fluorescence detector (Waters e2295-2475 FLR Detector, USA). Mobile phase A was 100% methanol. Mobile phase B consisted of 30 mM sodium acetate (pH 6.8).

### Transmission electron microscopy (TEM)

Rats were perfused transcardially with 2.5% glutaraldehyde following anesthetization. The PAG and its surrounding extensions were separated immediately after perfusion, then were post-fixed in 4% glutaraldehyde at 4°C for 24 h. Next, a sharp blade was used to cut the PAG tissues into 1 mm pieces, which was sent to Chongqing Medical University for subsequent fixation, embedding, slicing and staining according to the method as described previously (Wang et al., 2018). The images were taken using an EM-1400 PLUS transmission electron microscope. Statistical analysis was implemented with Image-pro Plus 6.2. The thickness of the postsynaptic density (PSD), the length of the synaptic activity zone, the width of the synaptic cleft and the curvature of the synaptic interface, four related indicators of the synaptic ultrastructure, were measured precisely. The length of the synaptic activity zone and the thickness of the PSD were detected based on the previous methods (Guldner and Ingham, 1980), the multipoint averaging method was performed to measure the width of the synaptic cleft and the curvature of the synaptic interface was measured according to the description of Jones and Devon (1978). The evaluations were performed by an observer blinded to the experimental groups.

### Golgi-Cox staining

The morphology of dendritic spines was analyzed by the FD Rapid Golgi Stain KitTM (FD NeuroTechnologies-Columbia, MD, USA). The PAG tissues were quickly isolated following rats were euthanized, and soaked in the mixture at room temperature in the dark for 2 weeks. The mixture was prepared with solution A and solution B at the 1:1 ratio, and it was refreshed once within 24 h. Then, the PAG tissues were transferred into solution C at room temperature in the dark for 3 days (the solution changed once after the first 24 h). The vibratome (Leica VT 1200S, Japan) was used to cut the PAG tissues into sections (150 μm thickness). Subsequently, in reference to the instructions of the kit, the sections were stained. After being sealed with a neutral resin, the dendritic spines of the PAG were imaged by a Zeiss microscope (Axio Imager A2) (Gibb and Kolb, 1998). Throughout the analysis, the experimenters were blinded to the experimental groups.

### Statistical analysis

All the data in this article represent the mean ± SD. Graphs were generated by GraphPad Prism 7. SPSS 20.0 was applied for statistical evaluations. All the data were tested for normality by the Kolmogorov-Smirnov (K-S) normality tests. Significant differences in pain thresholds were assessed using a two-way analysis of variance followed by a Bonferroni *post-hoc* test. Significant differences between the two groups were analyzed using independent-sample *t*-tests. A one-way analysis of variance followed by the Bonferroni *post-hoc* test was used to analyze multiple comparisons. A significance level of *p* < 0.05 was used.

## Results

### The inhibitory function of interneurons was insufficient in the PAG of CM rats

**FIGURE 2:**
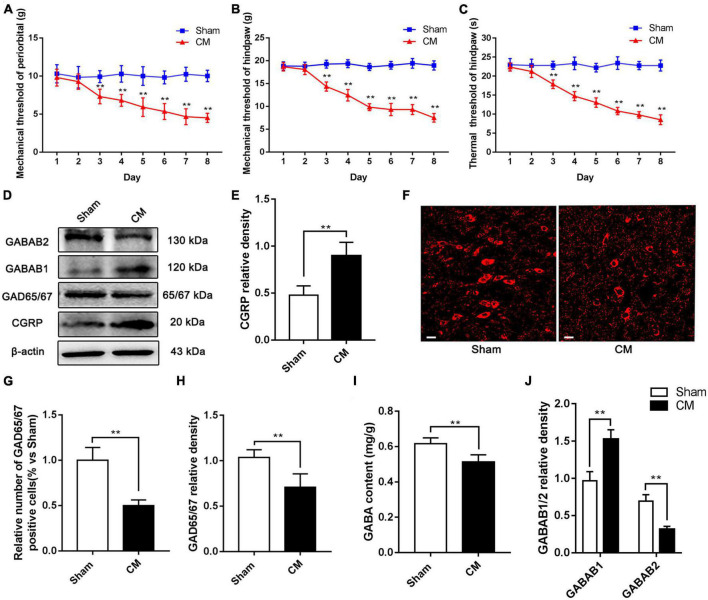
The inhibitory function of interneurons was insufficient in the PAG of CM rats. **(A,B)** Compared with the Sham group treated with PBS infusion, the mechanical pain thresholds in the periorbital region and the hindpaw in the CM group treated with IS infusion both significantly decreased after the second day (*n* = 10/group, **p < 0.01, vs. Sham). **(C)** The thermal pain thresholds in the hindpaw after the second day were significantly lower in the CM group than in the Sham group (*n* = 10/group, **p < 0.01, vs. Sham). **(D)** Representative western blottings of CGRP, GAD65/67, GABAB1 and GABAB2. **(E)** The expression level of CGRP was visibly increased in the CM group compared to the Sham group (*n* = 6/group, **p < 0.01). **(F)** Immunofluorescence staining of interneurons. **(G)** The number of interneurons was markedly decreased in the CM group than in the Sham group (*n* = 6/group, **p < 0.01). **(H,I)** Compared with the Sham group, the GAD65/67 and GABA expression levels in the CM group was substantially decreased (*n* = 6/group, **p < 0.01). **(J)** The protein level of GABAB1 in the CM group was greatly increased compared to the Sham group, while the protein level of GABAB2 was significantly lower in the CM group than in the Sham group (*n* = 6/group, **p < 0.01).

Calcitonin gene-related peptide (CGRP) is a classical biological marker of migraine (Edvinsson, 2017). In our study, we regarded CGRP as an important evaluation indicator of the CM state (Greco et al., 2022). To assess the reliability of the CM model, after the infusion of IS, we determined the mechanical pain threshold in the periorbital region, mechanical pain threshold in the hindpaw, thermal pain threshold in the hindpaw and CGRP expression level in the PAG. As shown in Figures 2A–C, beginning on the third day, we observed massive decreases in the mechanical and thermal pain thresholds in the IS group compared with the PBS group, suggesting that hyperalgesia occurred in CM rats. In addition, the expression level of GGRP was significantly increased in CM rats (Figures 2D, E). Taken together, these results show that the CM model was established successfully and is reliable.

To validate the dysfunction of inhibitory interneurons in CM, GAD65/67, a marker of inhibitory interneurons, was detected by immunofluorescence staining. As shown in Figures 2F, G, the relative number of inhibitory interneurons was markedly decreased in the CM group compared with the Sham group. In addition, the level of GAD65/67, the GABA synthase, was semiquantitatively determined by western blotting, and the results indicated that the GAD65/67 level in the CM group was significantly lower than that in the Sham group (Figures 2D, H). HPLC was used to measure the GABA content in the PAG, and the GABA content was lower in the CM group than in the Sham group (Figure 2I). GABABR is an important receptor for GABA, and the expression of the two subunits, GABAB1 and GABAB2, of GABABR was explored via western blotting. The protein level of GABAB1 in the CM group was greatly increased compared to that in the Sham group, while the protein level of GABAB2 in the CM group was significantly lower than that in the Sham group (Figures 2D, J). In summary, the above results reveal that the inhibitory function of interneurons is weakened in CM, which is consistent with our previous research (Zeng et al., 2020).

### Activation of GABABR elevated the pain thresholds in the periorbital region and suppressed the expression of CGRP in CM

**FIGURE 3:**
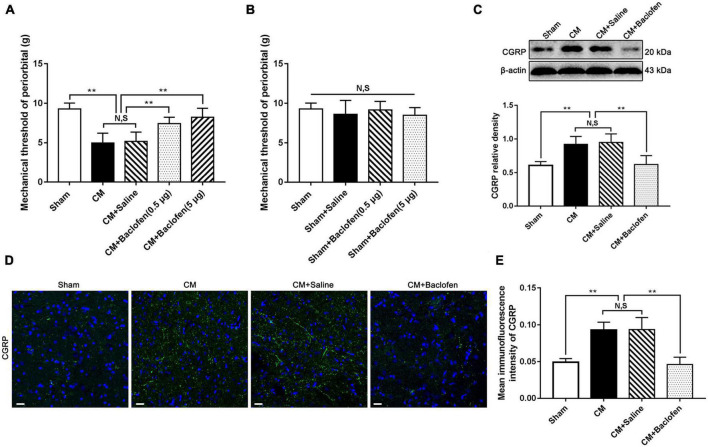
Effects of baclofen on pain thresholds of periorbital and CGRP expression level. **(A)** The mechanical pain thresholds in the periorbital region in the CM group were markedly lower than in the Sham group, and there was no significant difference between the CM and CM + Saline groups. Compared with the CM + Saline group, the mechanical pain thresholds in the periorbital region in the CM + baclofen (0.5 μg) and CM + baclofen (5 μg) groups both significantly improved (*n* = 10/group, **p < 0.01, N,S, not significant). **(B)** No significant difference was observed in the mechanical pain thresholds in the periorbital region among the Sham, Sham + Saline and Sham + baclofen groups (*n* = 10/group, N,S, not significant). **(C)** The protein level of CGRP in the CM group was substantially higher than in the Sham group, and there was no statistical difference between the CM and CM + Saline groups. The expression level of CGRP in the CM + baclofen group were markedly reduced compared to the CM + Saline group (*n* = 6/group, **p < 0.01, N,S, not significant). **(D,E)** The average fluorescence intensity of CGRP was drastically higher in the CM group than in the Sham group, and there was no significant difference between the CM and CM + Saline groups. Compared with the CM + Saline group, the average fluorescence intensity of CGRP substantially decreased in the CM + baclofen group (*n* = 6/group, **p < 0.01, N,S, not significant, scale bar = 20 μm).

To further explore the effects of dysfunction of inhibitory interneurons on synaptic plasticity mediated through GABABR in CM, we first injected the GABABR agonist baclofen into the lateral ventricle and tested the pain thresholds in the periorbital region in rats. As shown in Figure 3A, the pain thresholds in the periorbital region in the CM group were significantly lower than those in the Sham group. There was no difference between the CM and CM + Saline groups. Compared to those in the CM + Saline group, the pain thresholds in the periorbital region were markedly increased in the CM + baclofen (0.5 μg) and CM + baclofen (5 μg) groups. In addition, high-dose baclofen (5 μg) was applied in Sham rats to eliminate confounding from the possible toxic effects of baclofen. As shown in Figure 3B, there was no significant change in the pain thresholds in the periorbital region in any group, indicating that baclofen treatment had no effect on the pain thresholds in the periorbital region in the Sham group. Thus, high-dose baclofen (5 μg) was selected for subsequent studies.

Simultaneously, the expression of CGRP was detected by western blotting and immunofluorescence staining. The CGRP level in the CM group was significantly higher than that in the Sham group, and there was no substantial difference between the CM and CM + Saline groups. Treatment with baclofen markedly decreased the CM-evoked elevation of CGRP expression (Figure 3C). The results of the immunofluorescence analysis were fully consistent with those of western blotting (Figures 3D, E). Accordingly, these results indicate that GABABR signaling may play a vital role in CM and that the weakened function of inhibitory interneurons may contribute to the occurrence of CM through GABABR signaling.

### Activation of GABABR decreased the expression of the synapse-associated proteins PSD95, Syp and Syt-1 in CM

**FIGURE 4:**
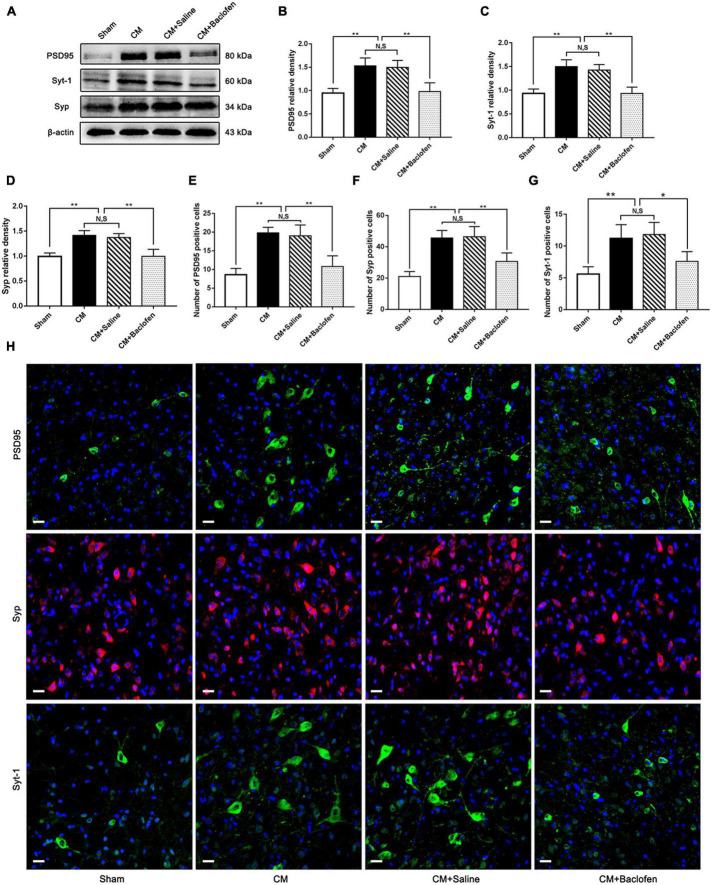
Effects of baclofen on the expression of the synapse-associated proteins PSD95, Syp and Syt-1. **(A)** Representative western blottings of PSD95, Syp and Syt-1. **(B–D)** The protein levels of PSD95, Syp and Syt-1 in the CM group were drastically higher than in the Sham group, and there was no significant difference between the CM group and the CM + Saline group. The PSD95, Syp and Syt-1 expression levels in the CM + baclofen group significantly reduced compared with those in the CM + Saline group (*n* = 6/group, **p < 0.01, N,S, not significant). **(E–G)** The numbers of PSD95, Syp and Syt-1 positive cells were substantially higher in the CM group than in the Sham group, and there was no statistical difference between the CM group and the CM + Saline group. Compared with the CM + Saline group. The numbers of PSD95, Syp and Syt-1 positive cells robustly decreased in the CM + baclofen group (*n* = 6/group, *p < 0.05, **p < 0.01, N,S, not significant, scale bar = 20 μm). **(H)** Immunofluorescence staining of PSD95, Syp and Syt. in the PAG.

To explore whether GABABR can modulate synaptic plasticity in CM, after the administration of baclofen, we measured the expression of the synapse-associated proteins PSD95, Syp and Syt-1 by western blot analysis. As shown in Figures 4A–D, the PSD95, Syp and Syt-1 levels were robustly increased in the CM group compared with the Sham group. No significant difference was found between the CM and CM + Saline groups. The PSD95, Syp and Syt-1 levels in the CM + baclofen group were significantly lower than those in the CM + Saline group. Then, we observed the changes in the numbers of PSD95-, Syp- and Syt-1-positive cells using immunofluorescence staining. As shown in Figures 4E–H, the numbers of PSD95-, Syp- and Syt-1-positive cells in the CM group were substantially elevated compared to those in the Sham group. There was no difference between the CM and CM + Saline groups. The application of baclofen significantly reduced the numbers of PSD95-, Syp-, and Syt-1-positive cells. Therefore, this expression pattern implies that GABABR may regulate synaptic plasticity in CM.

### GABABR regulated the synaptic ultrastructure and the number of dendritic spines in PAG neurons

**FIGURE 5:**
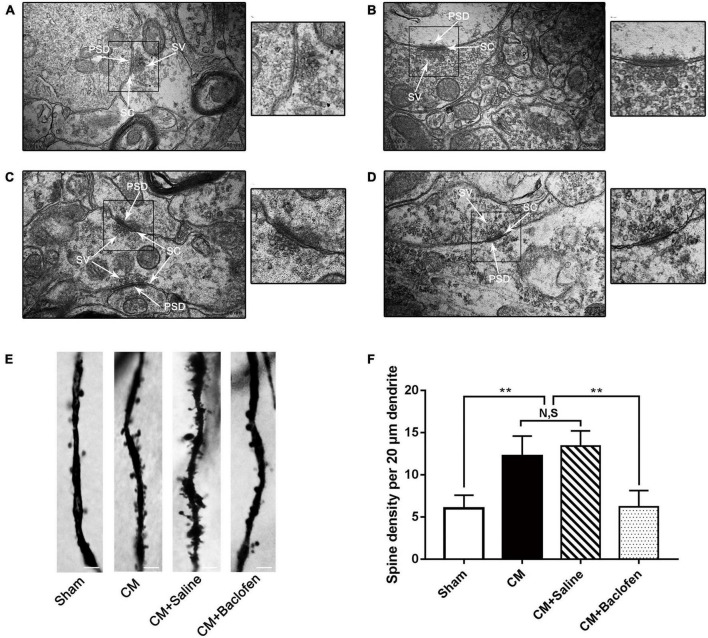
Changes in the synaptic ultrastructure and the density of dendritic spines in PAG neurons. **(A–D)** Synaptic ultrastructure in the four groups. A, a: Sham group; B, b: CM group; C, c: CM + Saline group; D, d: CM + baclofen group. PSD, postsynaptic density; SC, synaptic cleft; SV, synaptic vesicle. a–d show enlarged versions of the images in panels **(A–D)** (scale bars = 200 nm). **(E)** Representative photos of dendritic spines in the four groups. **(F)** Compared with the Sham group, the density of dendritic spines in the CM group was robustly elevated. GABABR activation by baclofen repressed the CM-induced increase of dendritic spine density, but there was no change between CM and CM + Saline groups (*n* = 6/group, **p < 0.01, N,S, not significant, scale bar = 5 μm).

The synaptic structure is the basis of synaptic plasticity (Xu-Friedman and Regehr, 2004). The synaptic ultrastructure of neurons in the PAG was observed under TEM following baclofen injection. Representative images are shown in Figures 5A–D. The synaptic contour in the Sham group was distinct and was confirmed by the clear presence of the presynaptic membrane, postsynaptic membrane and synaptic cleft. Abundant and transparent synaptic vesicles were observed in the anterior membrane region. However, indistinct synaptic clefts and presynaptic membranes were observed in the CM group, and all the morphological indicators of synapses, including the length of the active region, the curvature of the synaptic interface, the width of the synaptic cleft and the thickness of the postsynaptic density, were markedly increased in the CM group, indicating a higher synaptic transmission efficiency. No significant difference was observed between the CM and CM + Saline groups. Treatment with baclofen visibly reversed the CM-evoked changes in morphological indicators of synapses (Table 2). Overall, these results raise the possibility that GABABR may be involved in the modulation of synaptic plasticity in CM.

**TABLE 2:** Synaptic ultrastructure parameters in the PAG neurons in each group.

n = 5/group	Sham	CM	CM + Saline	CM + Baclofen
Thickness of the PSD/nm	17.0682 ± 0.45232	42.9048 ± 1.10979**	43.5972 ± 1.08955	26.8109 ± 1.15758^##^
Width of the synaptic cleft/nm	18.9064 ± 1.09413	32.4780 ± 1.19887**	32.6589 ± 1.13925	23.8603 ± 1.22111^#^
Length of active zones/nm	289.3919 ± 18.36563	474.0778 ± 15.45900**	472.2266 ± 16.61646	356.6438 ± 12.23606^##^
Curvature of synaptic interface	1.0696 ± 0.01701	1.2636 ± 0.01403**	1.2583 ± 0.01509	1.1673 ± 0.00502^##^

The thickness of the PSD, the width of the synaptic cleft, synaptic interface curvature and active zones in the CM group significantly increased compared with those in the Sham group, while treatment with baclofen reversed the changes. There was no statistical change between the CM and CM + Saline groups. Data are presented as the mean ± SEM (*n* = 6/group, **p < 0.01, vs. Sham group; ^#^*p* < 0.05, ^##^*p* < 0.01, vs. CM + Saline group).

Dendritic spines are actin-rich protrusions of the postsynaptic membrane, and they are crucial sites of excitatory synaptic input and vital components of synaptic function and plasticity (Elia et al., 2006; Mi et al., 2017). The dendritic spine density of pyramidal neurons was determined using Golgi-Cox staining in the PAG (Figures 5E, F). The density of dendritic spines in the CM group was substantially increased compared with that in the Sham group, and there was no difference between the CM and CM + Saline groups. Treatment with baclofen sharply decreased the density of dendritic spines. Thus, these results further support the idea that GABABR may regulate synaptic plasticity in CM.

### Activation of GABABR alleviated central sensitization in CM

**FIGURE 6:**
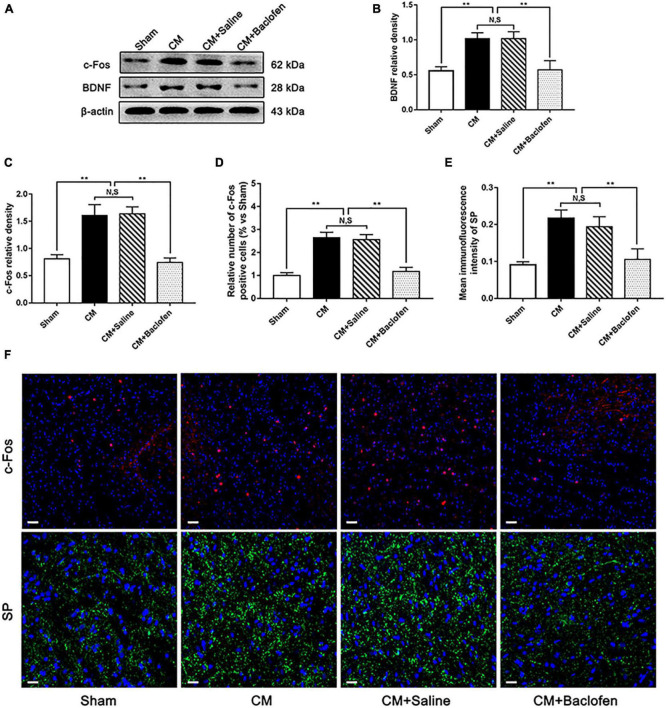
Effects of baclofen on the expression of BDNF, c-Fos and SP. **(A)** Representative western blottings of BDNF and c-Fos. **(B,C)** The protein levels of BDNF and c-Fos were significantly elevated in the CM group compared with those in the Sham group, and there was no statistical difference between the CM and the CM + Saline groups. The expression levels of BDNF and c-Fos in the CM + baclofen group robustly reduced compared to the CM + Saline group (*n* = 6/group, **p < 0.01, N,S, not significant). **(D,E)** The number of c-Fos positive cells and the average fluorescence intensity of SP in the CM group dramatically elevated compared to the Sham group, and there was no significant change between the CM and CM + Saline groups. The number of c-Fos positive cells and the average fluorescence intensity of SP in the CM + baclofen group were significantly lower than in the CM + Saline group (*n* = 6/group, **p < 0.01, N,S, not significant, scale bar = 20 μm). **(F)** Immunofluorescence staining of c-Fos and SP in the PAG.

To confirm whether GABABR is involved in central sensitization in CM, after the administration of baclofen, we determined the levels of the central sensitization-associated proteins BDNF, c-Fos and SP by western blotting or immunofluorescence assays (Wang et al., 2018; Long et al., 2020; Jiang et al., 2021). As shown in Figures 6A–C, the expression levels of BDNF and c-Fos were substantially higher in the CM group than in the Sham group. Their levels in the CM group were indistinguishable from those in the CM + Saline group. The levels of BDNF and c-Fos expression in the CM + baclofen group were dramatically decreased compared with those in the CM + Saline group. In addition, we observed changes in the number of c-Fos-positive cells and the average fluorescence intensity of SP by immunofluorescence analysis (Figures 6D–F). The number of c-Fos-positive cells and expression of SP in the CM group were markedly increased compared to those in the Sham group. There was no difference between the CM and CM + Saline groups. The application of baclofen robustly reduced the number of c-Fos-positive cells and expression of SP. Collectively, these observations reveal that GABABR may participate in central sensitization via the regulation of synaptic plasticity in CM.

### Activation of GABABR inhibited the pNR2B/CaMKII/pCREB pathway by the PKA/Fyn kinase (Fyn) axis in CM

**FIGURE 7:**
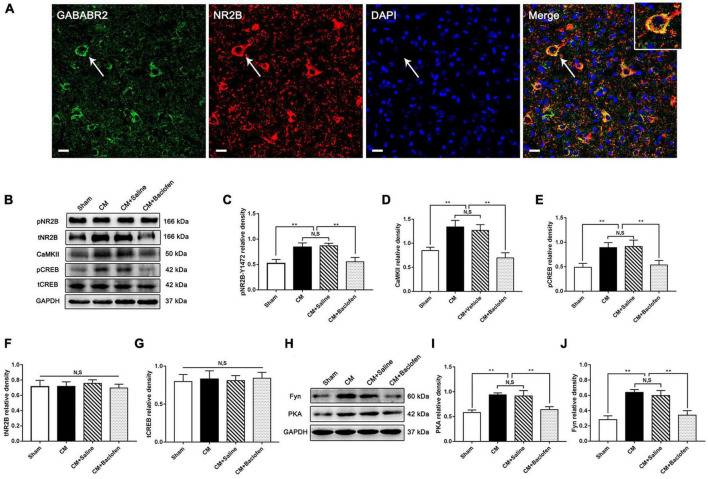
Effects of baclofen on the pNR2B/CaMK II/pCREB pathway and the PKA/Fyn axis. **(A)** Double immunofluorescence labeling of GABAB2 (green) and NR2B (red) revealed that GABAB2 and NR2B subunit co-expressed in the PAG (as shown by the white arrow) (*n* = 6/group, scale bar = 20 μm). **(B)** Representative western blottings of pNR2B, CaMK II, pCREB, total NR2B and total CREB. **(C–E)** The protein levels of pNR2B, CaMK II and pCREB in the CM group were drastically higher than in the Sham group, and there was no significant difference between the CM group and the CM + Saline group. The pNR2B, CaMK II and pCREB expression levels in the CM + baclofen group significantly reduced compared with those in the CM + Saline group (*n* = 6/group, **p < 0.01, N,S, not significant). **(F,G)** There was no significant difference in total NR2B and CREB expression among the Sham, CM, CM + Saline and CM + baclofen groups (*n* = 6/group, N,S, not significant). **(H)** Representative western blottings of PKA and Fyn. **(I,J)** The PKA and Fyn levels were significantly elevated in the CM group compared with those in the Sham group, and there was no statistical difference between the CM and the CM + Saline groups. The PKA and Fyn levels in the CM + baclofen group robustly reduced compared to the CM + Saline group (*n* = 6/group, **p < 0.01, N,S, not significant).

To investigate whether GABABR-regulated synaptic plasticity and central sensitization are related to NR2B in CM, we first used double immunofluorescence staining to explore the relationship between GABABR and the NR2B subunit in the PAG of Sham rats. We found that GABAB2 was coexpressed with the NR2B subunit (Figure 7A). Then, we used western blotting analysis to determine the protein levels of pNR2B/CaMKII/pCREB pathway components following the application of baclofen. As shown in Figures 7B–E, the protein levels of pNR2B, CaMKII and pCREB in the CM group were significantly higher than those in the Sham group. The levels of these proteins in the CM group were indistinguishable from those in the CM + Saline group. Treatment with baclofen drastically reduced the protein levels of pNR2B, CaMKII and pCREB. In addition, no differences in total NR2B and CREB levels were found among the Sham, CM, CM + Saline and CM + baclofen groups (Figures 7B, F, G).

To prove that GABABR regulates pNR2B through the PKA/Fyn axis, western blotting analysis was used to measure the expression of components of the PKA/Fyn axis. We observed that the expression of PKA and Fyn in the CM group was significantly higher than that in the Sham group. There was no difference between the CM and CM + Saline groups. Compared to that in the CM + Saline group, the expression of PKA and Fyn was markedly increased in the CM + baclofen group (Figures 7H–J). Collectively, the above results show that GABABR may modulate the pNR2B/CaMKII/pCREB pathway via the PKA/Fyn axis in CM.

### Inhibition of PKA suppressed the protein levels of Fyn and pNR2B in CM

**FIGURE 8:**
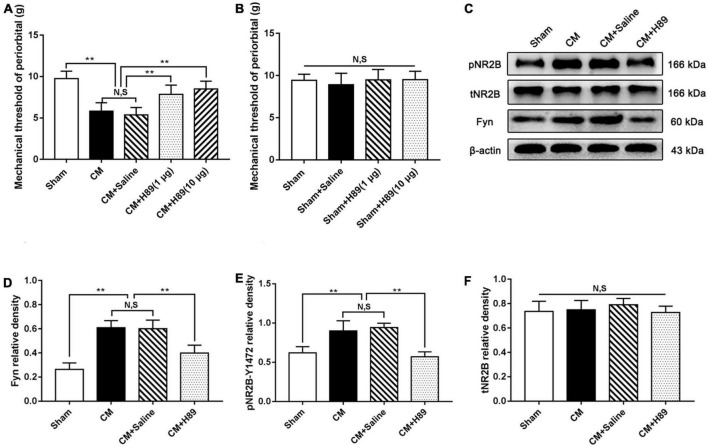
Effects of H89 on pain thresholds of periorbital and the levels of Fyn and pNR2B expression. **(A)** The mechanical pain thresholds of periorbital in the CM group were markedly lower than in the Sham group, and there was no significant difference between the CM and CM + Saline groups. Compared with the CM + Saline group, the mechanical pain thresholds of periorbital in the CM + H89 (1 μg) and CM + H89 (10 μg) groups both significantly improved (*n* = 10/group, **p < 0.01, N,S, not significant). **(B)** No significant difference was observed in the mechanical pain thresholds of periorbital among the Sham, Sham + Saline and Sham + H89 groups (*n* = 10/group, N,S, not significant). **(C)** Representative western blottings of Fyn, pNR2B and total NR2B. **(D,E)** The protein levels of Fyn and pNR2B in the CM group was substantially higher than in the Sham group, and there was no statistical difference between the CM and CM + Saline groups. The protein levels of Fyn and pNR2B in the CM + H89 group were markedly reduced compared to the CM + Saline group (*n* = 6/group, **p < 0.01, N,S, not significant). **(F)** There was no significant difference in total NR2B and CREB expression among the Sham, CM, CM + Saline and CM + baclofen groups (*n* = 6/group, N,S, not significant).

To convincingly verify that PKA regulates pNR2B via Fyn in CM, we injected the PKA inhibitor H89 into the lateral ventricle and measured the pain thresholds in the periorbital region in rats. As shown in Figure 8A, the pain thresholds in the periorbital region in the CM group were significantly lower than those in the Sham group. There was no difference between the CM and CM + Saline groups. Compared to those in the CM + Saline group, the pain thresholds in the periorbital region were dramatically increased in the CM + H89 (1 μg) and CM + H89 (10 μg) groups. In addition, high-dose H89 (10 μg) was applied in Sham rats to eliminate confounding from the possible toxic effects of H89. As shown in Figure 8B, no significant change in the pain thresholds in the periorbital region was found in any group, suggesting that H89 treatment has no effect on the pain thresholds in the periorbital region in the Sham group. High-dose H89 (10 μg) was selected for subsequent studies. Then, the protein levels of Fyn and pNR2B was measured by western blotting. The Fyn and pNR2B levels in the CM group were significantly higher than those in the Sham group, and there was no substantial difference between the CM and CM + Saline groups. Treatment with H89 markedly decreased the CM-evoked increases in the Fyn and pNR2B levels (Figures 8C–E). No change in the total NR2B level was observed in any group (Figures 8C, F). Accordingly, these results enhance the possibility that GABABR may modulate pNR2B via the PKA/Fyn axis.

## Discussion

Growing evidence suggests that the inhibition of interneurons plays a pivotal role in the manifestation of neuropathic and inflammatory pain states. The reduction in inhibitory interneurons due to cell death and the dysfunction of inhibitory interneurons can diminish their tone, which is followed by reduced release of GABA and inadequacy of GABABR-mediated inhibition, further altering their role in modifying synaptic transmission of pain regulation signals (Kim et al., 2012; Hogg et al., 2015; Bittar et al., 2017), particularly in the primary afferent terminals, which thus induces spontaneous pain behavior or hyperalgesia (Engle et al., 2006; Polgar and Todd, 2008). Analogously, we found reductions in the number of interneurons and content of GABA in CM, indicating dysfunction of inhibitory interneurons in CM. GABABR is a heterodimer, and GABAB1 and GABAB2 can be assembled into the functional GABABR complex. GABAB1 is responsible for binding to extracellular ligands, while GABAB2 is mainly engaged in transducing intracellular signals. GABAB2 can promote the translocation of GABAB1 to the cell surface, stabilize the receptor, and bind to the inhibitory Gi/o protein (Enna, 2001; Kubo and Tateyama, 2005). The two subunits have a clear division of labor, and GABABR cannot perform its inhibitory function without either of the two subunits. In this study, GABAB1 expression was increased but GABAB2 expression was decreased in CM rats compared with sham rats, suggesting that the number of fully functional GABABR complexes assembled from GABAB1 and GABAB2 in CM may be reduced due to the decrease in GABAB2. Thus, dysfunction of inhibitory interneurons and the decreased GABABR level cooperatively lead to a deficit in the inhibitory intensity in CM. Considering the occurrence of hyperalgesia in CM, it is tempting to speculate that the loss of inhibitory interneuron-mediated inhibition appears to be involved in regulating nociceptive signaling. This scenario is supported by the finding that treatment with baclofen drastically alleviated CM-induced hyperalgesia. Baclofen is the most common GABABR agonist, and after GABAB1 binds to baclofen, activated GABAB2 further amplifies signaling through G protein signal transduction, thereby exerting a slow and persistent inhibitory effect (Kent et al., 2020) (this is the activation process of baclofen to GABABR), and finally alleviating CM.

Phosphorylation of NMDA receptors is the crucial molecular mechanism underlying central sensitization (Liu and Salter, 2010). Repressing NMDA receptors has been broadly studied in terms of weakening excitatory synaptic transmission in diverse neurological diseases (Ali and Salter, 2001; Wood, 2005). NR2B is an extensively studied protein of the NMDA receptors, and the maintenance of NMDAR receptor activation may depend on the pNR2B-mediated localization and trafficking in synapses (Qiu et al., 2011). NR2B phosphorylation has important consequences for the process of chronic pain (Qiu et al., 2011). Similarly, in the PAG, the level of phosphorylated NR2B was significantly increased in CM rats, indicating the overactivation of NMDA receptors in CM. After baclofen was injected into the lateral ventricle in the CM group, we observed that activation of GABABR resulted in a decline in NR2B phosphorylation in the PAG, which proves that GABABR may regulate pNR2B activity. Many studies have shown that the activation of GABABR suppresses PKA activity by modulating adenylyl cyclase (AC)/cAMP signaling (Zhu Y. S. et al., 2019), PKA can activate the Src-family protein tyrosine kinase member Fyn, possibly by disrupting the association of Fyn with its inhibitory partner striatal-enriched protein tyrosine phosphatase 61, and active Fyn then promotes NR2B phosphorylation at Tyr1472 (Yang et al., 2011; Qiu et al., 2013). Consistent with this observation, in our study, activation of GABABR or inhibition of PKA inhibited the CM-evoked elevation of Fyn and pNR2B protein levels in rats. In brief, these results support the speculation that the loss of inhibitory interneuron function regulates pNR2B-mediated nociceptive signaling in CM and that this process may be related to the GABABR/PKA/Fyn/pNR2B pathway. GABABR-evoked modulation of NR2B phosphorylation is a contributing factor to the development of CM.

In different areas of the central nervous system, the necessity of the NR2B subunit of NMDA receptors in terms of synaptic potentiation has been proven (Zhuo, 2009). In the anterior cingulate cortex, the administration of selective NR2B antagonists markedly inhibited but did not completely block the induction of LTP (Zhao et al., 2005). Likewise, in the lateral amygdala, NMDA receptor-dependent LTP substantially declined after the application of the NR2B antagonist Ro25-6981 (Miwa et al., 2008). Consistent with this observation, the regulation of NR2B phosphorylation in the synaptic plasticity of CM was also demonstrated in our previous study (Wang et al., 2018). To further explore whether the regulation of NR2B phosphorylation by GABABR may alter synaptic plasticity in CM, we determined the expression of three synapse-related indicators PSD95, Syp and Syt-1 to emphasize changes in synaptic plasticity. Our data revealed that the expression levels of PSD95, Syp and Syt-1 in the PAG of CM rats were robustly increased and then sharply declined under baclofen treatment, implying that the expression of synaptic function/structure-related proteins is closely correlated with activation of GABABR in the PAG and that GABABR may have regulatory effects on synaptic plasticity by modulating NR2B phosphorylation. These views are further strongly supported by the results of TEM and Golgi-Cox staining. The thickness of the PSD, the length of the synaptic activity zone, the width of the synaptic cleft and the curvature of the synaptic interface are four related indicators of synaptic ultrastructure. Increases in these parameters are the most intuitive manifestation of the increase in synaptic transmission efficiency in CM, and baclofen treatment reversed their elevation. Similarly, the increase in the intensity of dendritic spines induced by CM was also reversed by GABABR activation. These results further confirm that inadequacy of interneuron-related inhibition may modulate synaptic plasticity via GABABR-pNR2B signaling.

Brain-derived neurotrophic factor is a member of the neurotrophin class of growth factors (Nijs et al., 2015). Modulation of TrkB receptor-related BDNF signaling is recognized as a key culprit responsible for neuropathic pain (Constandil et al., 2011; Richner et al., 2019). In CM, we have obtained reliable evidence that BDNF regulates glutamate input to neurons, elevating the excitability of neurons and thereby participating in central sensitization (Liu et al., 2018; Long et al., 2020). The nuclear protein c-Fos, encoded by the immediate early gene c-fos, is rapidly expressed in response to different types of noxious stimuli in neurons (He et al., 2019). Furthermore, SP participates in the modulation of nociceptive transmission and is vital in migraine pathophysiology (Fusayasu et al., 2007), and the release of SP is proportional to the intensity and frequency of the nociceptive stimulus (Mantyh, 2002). In our research, we found significant increases in the expression levels of BDNF, c-Fos and SP in the PAG of CM rats, which was likely linked to the enhancement of the processing of nociceptive information. Activation of GABABR markedly reversed the CM-evoked upregulation of BDNF, c-Fos and SP, indicating that GABABR-regulated synaptic plasticity may play a decisive role in central sensitization in CM. CaMKII, a serine/threonine protein kinase, can be activated by NMDA receptor-mediated Ca^2+^ influx; CaMKII is an important molecule in neural plasticity and memory (Zalcman et al., 2018), and CaMKII/CREB signaling is involved in the development of some types of chronic pain (Zhou et al., 2017). While CREB phosphorylation at Ser133 increases the expression levels of the nociceptive neurotransmitters CGRP, BDNF, and SP by enhancing their transcription levels in the nucleus (Wan et al., 2016; Xie et al., 2018; Lu et al., 2021), the release of these nociceptive neurotransmitters activates neurons, thus increasing the expression of c-Fos (Mitsikostas et al., 2011). Analogously, we observed that the pNR2B/CaMKII/pCREB pathway was overactivated in CM, that the application of baclofen suppressed this pathway, and that the expression of nociceptive neurotransmitters and neuronal activation were ultimately affected by regulation of this pathway. Collectively, the expression patterns further enhance the possibility that deficiency of inhibitory interneuron function may facilitate central sensitization by regulating GABABR-pNR2B signaling in CM rats.

**FIGURE 9:**
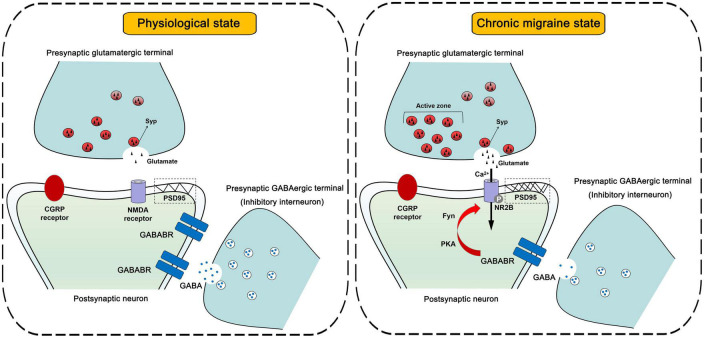
Schematic diagrams of the inhibitory interneurons-mediated regulation of synaptic plasticity in central sensitization.

In brief, we propose a novel perspective on the function of inhibitory interneurons involved in the regulation of synaptic plasticity in CM (Figure 9) and pay close attention to the role of the GABABR-regulated synaptic plasticity mechanism in the development of central sensitization in CM, which facilitates the further discovery of inhibitory system-related signaling molecules as promising candidates for the future treatment or even prevention of CM.

## Data availability statement

The original contributions presented in this study are included in the article/Supplementary material, further inquiries can be directed to the corresponding author.

## Ethics statement

This animal study was reviewed and approved by the Ethics Committee of the Department of Medical Research (First Affiliated Hospital of Chongqing Medical University).

## Author contributions

LC and XZ designed the study. XZ and YN performed the experiments and wrote the manuscript. XZ analyzed the data. GQ and DZ provided the valuable advice on the design of this study and image modification. All authors read and approved the final manuscript.
