# Supplementary material for: Dysfunction of inhibitory interneurons contributes to synaptic plasticity via GABABR-pNR2B signaling in a chronic migraine rat model
Source: Front Mol Neurosci. 2023 May 26;16:1142072. doi: 10.3389/fnmol.2023.1142072 (PMC10265202; doi:10.3389/fnmol.2023.1142072)
Supplement: Supplementary file 1 [file Data_Sheet_1.DOCX]

Dear editor,

All the original images involved in our manuscript have been uploaded: <https://www.jianguoyun.com/p/DW_0jKYQ2_OqCxjR2vIEIAA.>

If you have any questions please feel free to contact us.

Sincerely,

Lixue Chen
chenlixue@hospital.cqmu.edu.cn
